# Moringa isothiocyanate-1 inhibits LPS-induced inflammation in mouse myoblasts and skeletal muscle

**DOI:** 10.1371/journal.pone.0279370

**Published:** 2022-12-16

**Authors:** Badi Sri Sailaja, Sohaib Hassan, Evan Cohen, Irina Tmenova, Renalison Farias-Pereira, Michael P. Verzi, Ilya Raskin

**Affiliations:** 1 Department of Plant Biology, School of Environmental and Biological Sciences, Rutgers, The State University of New Jersey, New Brunswick, New Jersey, United States of America; 2 Department of Genetics and the Human Genetics Institute of New Jersey, Rutgers, The State University of New Jersey, Piscataway Township, New Jersey, United States of America; Tohoku University, JAPAN

## Abstract

This study aims to investigate the anti-inflammatory effects of moringa isothiocyanate-1 (MIC-1) extracted from seeds of *Moringa oleifera* Lam. in lipopolysaccharide (LPS)-induced inflammation models. MIC-1 decreased nitric oxide production and reduced the expression of pro-inflammatory markers (TNF-α, Ifn-α, IL-1β, IL-6) in C2C12 myoblasts. The daily oral treatment of MIC-1 (80 mg/kg) for three days significantly reduced the expression of pro-inflammatory markers in gastrocnemius muscle tissue of LPS-treated C57BL/6 male mice. Transcriptomic analysis provided further insights into the inhibitory effects of MIC-1 on the LPS-induced inflammation, which suggested that MIC-1 affects inflammation and immunity-related genes in myoblasts and skeletal muscle tissue. MIC-1 inhibited the nuclear accumulation of the nuclear factor kappa-light-chain-enhancer of activated B cells (NF-κB) in the LPS-treated myoblasts. Our data support the hypothesis that the MIC-1’s effects in the muscle cells are mediated through the inhibition of the NF-κB translocation in the nucleus, which, in turn, results in immunomodulating and anti-inflammatory responses at the gene expression levels.

## Introduction

Moringa isothiocyanate-1 (MIC-1, also known as moringin) is an isothiocyanate that has similar biological activities as other natural isothiocyanates, such as sulforaphane from cruciferous vegetables. Isothiocyanates are the class of plant secondary metabolites originating from the sulfur-containing compounds glucosinolates. MIC-1 is found in *Moringa oleifera* Lam., a plant used as food and medicine, with concentrations up to 40% in the hydro-alcoholic moringa seed extract [1]. In contrast to liquid sulforaphane, MIC-1 contains a sugar group that makes it a stable solid at room temperatures [2]. MIC-1’s chemical structure also improves its in vivo stability and bioavailability [2, 3]. Sixty percent of MIC-1, applied as pure compound or as a component of moringa seed extract, was not degraded in the in vitro gastrointestinal digestion model [3]. Orally administered MIC-1, up to 100 mg/kg bw/d, did not cause toxic effects in rats [4]. These properties make MIC-1 a potentially useful addition to the human diet.

Previous reports showed that MIC-1 has anti-inflammatory, antioxidant, and antimicrobial activities [1, 2, 5–8]. The biological activities of moringa isothiocyanates, including MIC-1, have been recently reviewed by Lopez-Rodriguez et. al. [9]. In vitro studies showed that MIC-1 inhibits pro-inflammatory pathways [1, 7, 8, 10–14], and reduces lipopolysaccharide (LPS)-induced inflammation by inhibiting nuclear factor kappa B (NF-κB) pathway in macrophages [8]. NF-κB is a transcription factor that induces inflammation by upregulating cytokines, such as the tumor necrosis factor alpha (TNF-α), interleukins (IL), and interferons (Ifn). Consistently, previous studies have shown that MIC-1 decreases pro-inflammatory cytokines (TNF-α, IL-6, IL-1β, and Ifn-α) in macrophages [2, 7, 8, 10–12]. MIC-1 reduced the production of nitric oxide (NO), a pro-inflammatory marker, by downregulating the inducible nitric oxide synthase (iNOS) gene in LPS-treated macrophages [1]. MIC-1 inhibited inflammatory pathways, including LPS, NF- κB, IL-1, NO signaling pathways in skin carcinogenesis and nephropathy models in vitro as well [13, 14].

The anti-inflammatory mechanisms of MIC-1 have also been investigated by using in vivo models. We previously showed that the daily oral supplementation with MIC-1 (80 mg/kg bw) for 3 days protects from systemic inflammation induced by LPS in mice [8]. We used transcriptomics analyses and targeted molecular biology to show that MIC-1 downregulated inflammatory pathways and pro-inflammatory cytokines reversing the LPS-induced inflammatory response in spleen, liver, kidney and colon [8]. Others have shown that MIC-1 (10 mg/kg bw; ip) reduced pro-inflammatory cytokines in brain in a murine model for Parkinson’s disease [10], and the transdermal application of 2% MIC-1, as a cream, had anti-inflammatory effects in spinal cord tissue in a murine model for multiple sclerosis [15]. Other studies have suggested that MIC-1, administered in moringa extracts, reduces inflammatory markers in blood, adipose tissue, liver, and intestine in mice with diet-induced metabolic dysfunction [6, 16]. Although there is evidence that MIC-1 acts as anti-inflammatory compound in several models, there is no report that investigated if MIC-1’s anti-inflammatory effects extend to skeletal muscle.

Since MIC-1 and other isothiocyanates have anti-inflammatory properties, MIC-1 may be used to promote skeletal muscle health. Pre-clinical and clinical studies have suggested that sulforaphane ameliorates muscle damage caused by inflammation [17–20]. Sulforaphane inhibited NF- κB pathway, including the levels of IL-1β and IL-6, in a genetic model for muscle dystrophy [17]. Also, MIC-1 may improve skeletal muscle health due to its antioxidant effects, as seen in moringa leaf extracts-treated C2C12 cells [21–24]. For instance, moringa leaf extract reduced oxidative stress induced by hydrogen peroxide in C2C12 myotubes [24]. Skeletal muscle plays a major role in a host response to sepsis contributing to signaling pathways of inflammation and metabolic dysfunctions [25, 26]. Since others have shown that both C2C12 myoblasts in vitro and gastrocnemius muscle in vivo are responsive to LPS-induced inflammation by increasing the pro-inflammatory cytokines, such as TNF-α and IL-6 [27], we decided to evaluate the effects of MIC-1 on LPS-induced inflammation in mouse myoblasts and gastrocnemius muscle tissue. Our current study confirms that MIC-1 reduces LPS-induced muscle inflammation by downregulating pro-inflammatory cytokines via NF-κB pathway, and MIC-1 likely inhibits other inflammation-associated muscle disorders as well.

## Materials and methods

### Materials

MIC-1 (purity >98%) was isolated from *Moringa oleifera* seed extract as previously reported [1]. All other materials were purchased from commercial suppliers. C2C12 cells were obtained from American Type Culture Collection (ATCC, Manassas, VA, USA). C57BL/6 male mice were acquired from Charles River Laboratories (Malvern, PA, USA). Cell culture materials: Dulbecco’s modified Eagle’s medium (DMEM), penicillin G, streptomycin, and fetal bovine serum (FBS) were obtained from Gibco Inc. (Grand Island, NY). 3-(4,5-Dimethylthiazol-2-yl)-2,5-diphenyltetrazolium bromide (MTT) was obtained from Cayman Chemical (Ann Arbor, MI, USA) and Griess reagent system was acquired from Promega Corporation (Madison, WI, USA). Lipopolysaccharide (LPS, Escherichia coli 0111: B4), dimethyl sulfoxide (DMSO), and other chemicals were purchased from Sigma (St. Louis, MO, USA). Gene probes were obtained from Integrated DNA Technologies (Coralville, IA, USA), and the probe sequences are the same as our previous study [8]. RNeasy Mini Kit was obtained from Qiagen (Germantown, MD, USA). Anti-NF-κB p65 antibody (ab16502) was obtained from Abcam (Cambridge, MA, USA), and 4′,6-diamidino-2-phenylindole (DAPI) and Alexa Fluor™ 680 phalloidin were purchased from Invitrogen (Carlsbad, CA, USA).

### In vitro assays

C2C12 myoblasts were cultured in a growth medium (90% DMEM, 10% FBS, 100 units/mL penicillin, and 100 μg/mL streptomycin) at 37°C in a humidified atmosphere with 5% carbon dioxide. MIC-1 solutions were made in ethanol. Myoblasts were treated with 0.475% ethanol (vehicle), or 1–10 μM MIC-1, with or without 1 μg/mL LPS for 24 h before the in vitro assays.

Cell viability was measured by using MTT, which was added to the growth medium to create purple formazan crystals [1]. Absorbance values were obtained by spectrophotometric measurements at 570 nm, and values were reported as % of control (vehicle).

Nitric oxide (NO) production was evaluated by using Griess method [1]. The concentrations of NO in the cell media were calculated by using nitrite as standard. Absorbance was read at 550 nm, and values were reported as % of LPS-treated group.

Immunofluorescence staining was used for NF-κB nuclear translocation assessment as previously described [8]. Fixed cells were stained with anti-NF-κB p65 antibody with fluorescent counterstains for F-actin and DAPI. The percentage of nucleic positive cells to NF-kB (p65) was quantified by counting 100 cells per field in three separate experiments [28].

### Animals and tissue collection

Eighteen C57BL/6 male mice were randomly separated into vehicle control, LPS-treated, or LPS-MIC-1-treated group as previously described [8]. MIC-1 (80 mg/kg bw) was orally administered by gavage daily for 3 days before LPS treatment (10 mg/kg bw; ip). Gastrocnemius muscles were collected from euthanized mice after 16 h of LPS treatment. Fat and connective tissues were trimmed from muscles before freezing in liquid nitrogen for further analyses. The handling and care of animals used in this study followed the standard procedures recommended by the Guide for the Care and Use of Laboratory Animals of the National Institutes of Health. All efforts were made to minimize suffering and distress. No animal showed behavioral and physiological signs of pain and distress including changes in feeding pattern, body temperature, and social interaction after 16 h of LPS treatment. Animals were sacrificed by carbon dioxide asphyxiation. The protocol was approved by the Rutgers University Institutional Animal Care and Use Committee (ID999900474).

### RNA sequencing and analysis

RNA-sequencing data (Gene Expression Omnibus accession #GSE209706) were analyzed by using the transcript abundances from pseudoalignments in Kallisto (v0.46.11), an RNA-seq quantification program [8, 29]. The differentially expressed genes were indicated by the normalized fragments per kilobase reads per million (FPKM) values. FPKM values (> 1) were analyzed by using tximport package (v 1.18.02) in R (v 4.0.3), and DESeq2 (v 1.30.13) to determine the differences in gene expression (*P*-value < 0.05, or < 0.001; absolute log2-fold change > 1; FPKM > 1) [30, 31]. Morpheus heat mapping software (Broad Institute) was used to illustrate the identified clusters of differentially expressed genes by using k-means clustering analysis. Gene Ontology (GO) analysis by the Database for Annotation, Visualization and Integrated Discovery (DAVID, v6.84) was used to identify the biological processes of genes, expressed as negative log10 (*P*-values). Reproducibility of RNA-seq data is shown in principal component analysis (PCA) plots (S1 Fig); DESeq2 analysis of triplicates for C2C12 samples and muscle tissue samples was conducted to create PCA plots. The data were first turned into a DESeq2 object, then ’regularized log’ transformed and plotted in a PCA plot.

### Gene expression

Reverse transcription quantitative real-time PCR was performed to measure the expression of pro-inflammatory genes as previously described [8]. Power SYBR Green PCR Master Mix was used to detect the amplification of the target genes by using the QuantStudio™ 3 Real-Time PCR System (Applied Biosystems, Waltham, MA, USA). GAPDH gene expression was used as housekeeping gene to normalize the expression of target genes.

### Statistical analysis

Data are expressed as mean ± standard deviation (SD) of three experiments in triplicate. One-way analysis variance or Student’s t-test were used to determine statistical differences (*P* < 0.05) by using GraphPad (GraphPad, La Jolla, CA).

## Results

### Effect of MIC-1 on LPS-induced inflammation in C2C12 murine muscle cells

**Fig 1:**
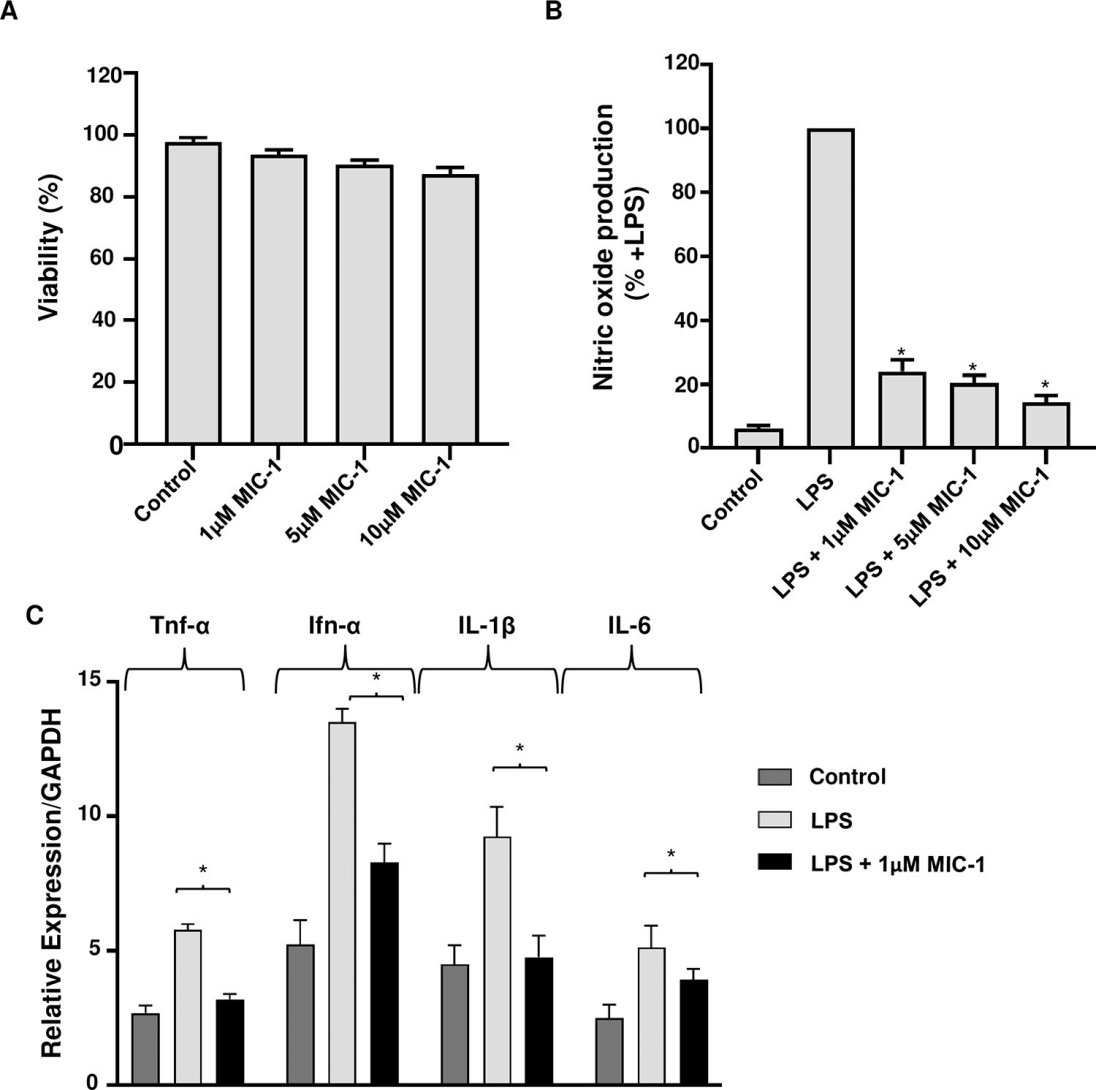
Effect of moringa isothiocyanate-1 (MIC-1) in C2C12 myoblasts. Cells were treated with MIC-1 (1, 5 or 10 μM), or 0.475% ethanol (vehicle), with or without 1 μg/mL lipopolysaccharide (LPS) for 24 h. (A) Cell viability. (B) Nitric oxide (NO) production. (C) Gene expression of pro-inflammatory markers (TNF-α, Ifn-α, IL-1β and IL-6). Data are represented as mean ± S.D. (n = 3). * *P* < 0.05, obtained by t-test when compared to LPS-treated group.

To assess the cytotoxicity of MIC-1, we performed a cell viability assay in C2C12 murine cells treated with MIC-1 (1, 5 or 10 μM). MIC-1 did not significantly affect the cellular viability at the tested concentrations (Fig 1A). Next, we evaluated the effects of MIC-1 on NO production with LPS (1 μg/mL) and found a significant dose dependent reduction in NO production (Fig 1B). Furthermore, we found that 1 μM MIC-1 downregulated significantly the pro-inflammatory genes TNF-α, Ifn-α, IL-1β, and IL-6 (Fig 1C). These results suggested that MIC-1 inhibits LPS-induced inflammation without major effects on the viability of muscle cells.

### Transcriptome analysis of MIC-1 in LPS-induced inflammation muscle cells

**Fig 2:**
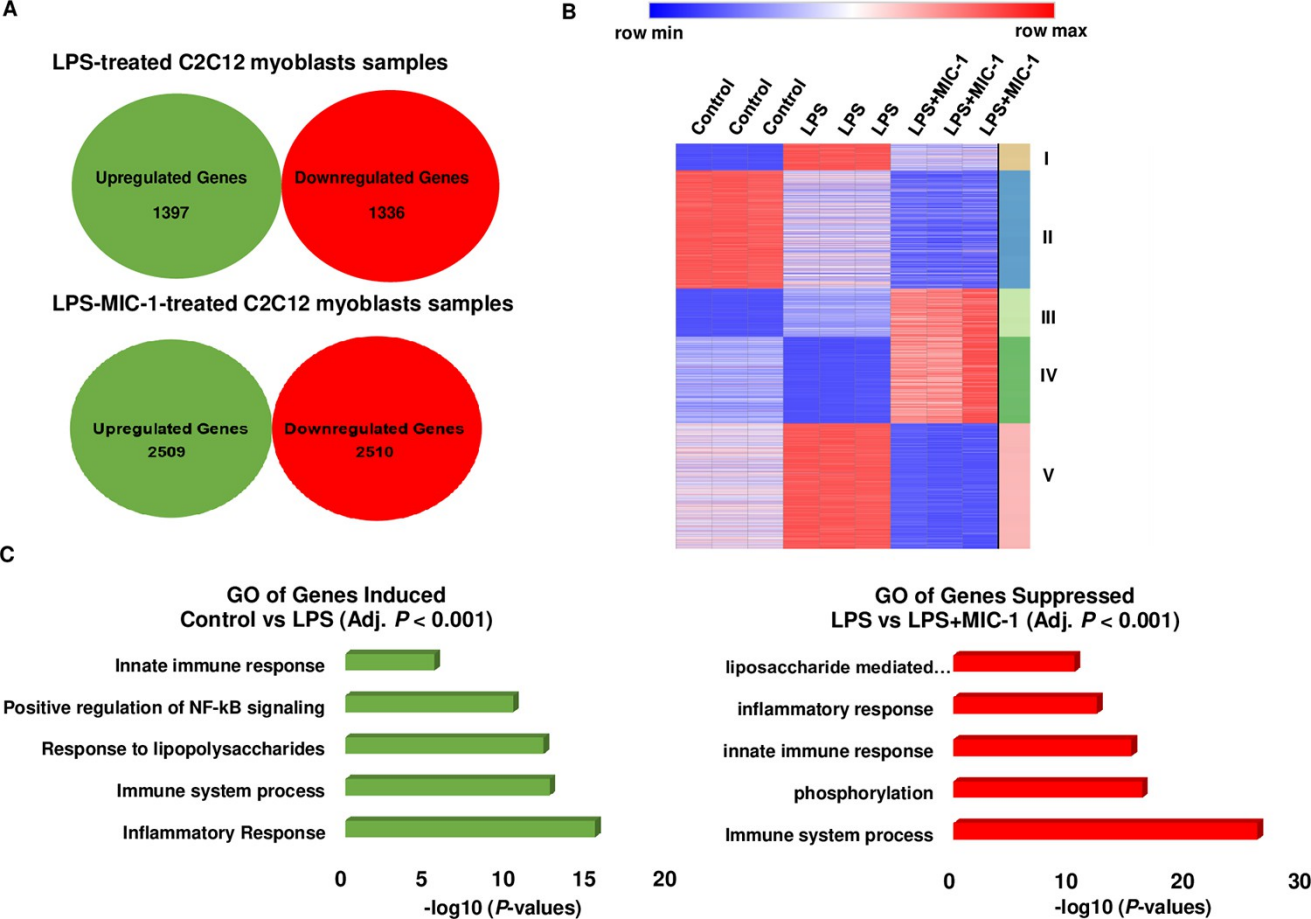
Transcriptome analysis of moringa isothiocyanate-1 (MIC-1) in C2C12 myoblasts. (A) Venn diagrams depict genes significantly (absolute log2-fold change > 1; Adj. *P* < 0.001) upregulated/downregulated due to LPS (control vs. lipopolysaccharide (LPS)-treated samples) or MIC-1 (LPS-treated samples vs. LPS-MIC-1-treated samples) in C2C12 murine muscle cells. (B) Heat map of differentially expressed genes in five k-means clusters. Column, row, and color represent a sample, gene, and expression level, respectively. (C) Gene ontology (GO) analysis of upregulated genes due to LPS (control vs. LPS-treated samples) and downregulated genes due to MIC-1 (LPS-treated samples vs. LPS-MIC-1-treated samples), represented as negative log10 (*P*-values).

Transcriptomic analysis of 1 μM MIC-1 and LPS-treated myoblasts was performed to evaluate the effects of MIC-1 on inflammation in C2C12 skeletal muscle cells. Control samples were compared to LPS-treated samples to assess the effects of LPS, and LPS-treated samples were compared to LPS-MIC-1-treated samples to assess the effects of MIC-1 on the LPS-induced inflammation. As shown in Fig 2A, 1397 genes were upregulated, and 1336 genes were downregulated due to LPS treatment (control vs LPS-treated samples). A total of 2509 genes were upregulated, and 2510 genes were downregulated due to MIC-1 treatment (LPS-treated samples vs LPS-MIC-1-treated samples) (Fig 2A). The differentially expressed genes (adjusted *P*-value < 0.001 and FPKM > 1), identified by DESeq2 analysis using the pairwise comparisons (control vs LPS-treated samples; LPS-treated samples vs LPS-MIC-1-treated samples), were further clustered by using k-means to identify clusters of genes showing common responses to these treatments. This list of 3,567 genes were clustered in five clusters (Fig 2B). Cluster V was particularly interesting, as 1,104 genes were induced by LPS and subsequently repressed by the MIC-1 treatment (Fig 2B). GO analysis of these genes revealed that functions such as innate immune response, inflammatory response, and immune system processes were induced by LPS, and subsequently repressed by MIC-1 (Fig 2C; S1 File). These results suggest that the anti-inflammatory properties of MIC-1 in the LPS-treated myoblasts result from a direct effect of MIC-1 on the inflammation and immunity pathways.

### Transcriptome analysis of MIC-1 effect in the LPS-induced muscle tissue

**Fig 3:**
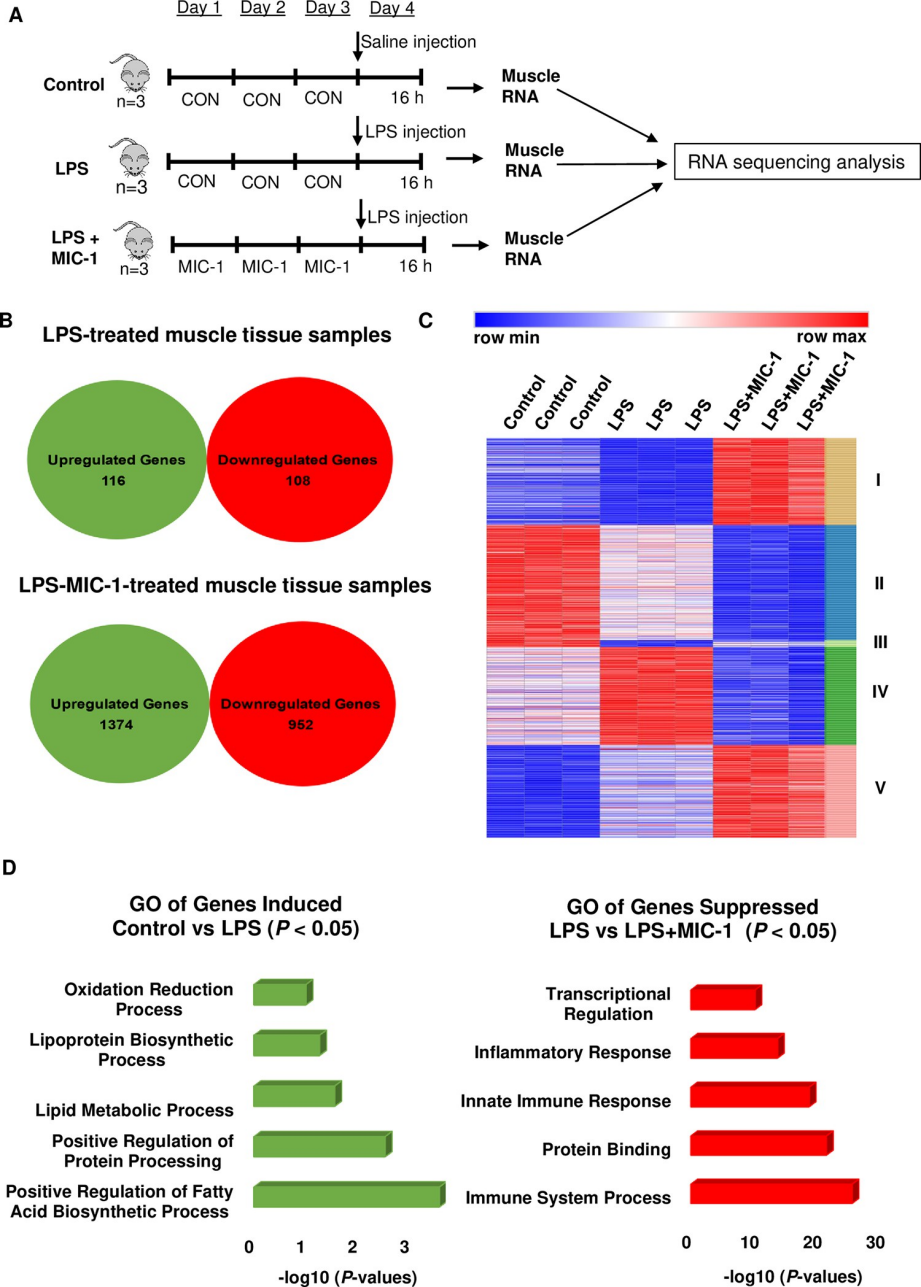
Transcriptome analysis of moringa isothiocyanate-1 (MIC-1) in muscle tissue. (A) Schematic diagram of the experimental protocol. MIC-1 was given orally daily for 3 days to C57BL/6 male mice before lipopolysaccharide (LPS) treatment. Mice were randomly divided into three groups: control (10% DMSO; p.o.); LPS (10 mg/kg bw; ip); LPS + MIC-1 (80 mg/kg bw; p.o.). Muscle tissues were collected 16 h after LPS treatment (n = 3). (B) Venn diagrams depict genes significantly (absolute log2-fold change > 1; *P* < 0.05) upregulated/downregulated due to LPS (control vs. LPS-treated samples) or MIC-1 (LPS-treated samples vs. LPS-MIC-1-treated samples) in murine gastrocnemius tissues. (C) Heat map of differentially expressed genes in five k-means clusters. Column, row, and color represent a sample, gene, and expression level, respectively. (D) Gene ontology (GO) analysis of upregulated genes due to LPS (control vs. LPS-treated samples), and downregulated genes due to MIC-1 (LPS-treated samples vs. LPS-MIC-1-treated samples), represented as negative log10 (*P*-values).

MIC-1 (80 mg/kg bw) was orally administered to C57BL/6 male mice for three days before LPS treatment (10 mg/kg bw; ip). Thereafter, the gastrocnemius muscle tissues of animals were collected 16 h after the LPS injection for transcriptomic analyses by RNA-seq (Fig 3A). We compared untreated control vs LPS-treated samples, and LPS-treated samples vs LPS-MIC-1-treated samples to understand the response to LPS and its attenuation by MIC-1 in the skeletal muscle tissue. Our results showed that 116 genes were upregulated due to LPS (control vs LPS-treated samples) (Fig 3B). A total of 1374 genes were upregulated, and 952 genes were downregulated due to MIC-1 (LPS-treated samples vs LPS-MIC-1-treated samples) (Fig 3B). The differentially expressed genes (adjusted *P*-value < 0.05), identified by DESeq2 analysis using the pairwise comparisons (control vs LPS-treated samples; LPS-treated samples vs LPS-MIC-1-treated samples), were further clustered by using k-means to identify clusters of genes showing common responses to these treatments. Five k-means clusters were obtained from this list of 1,049 genes (Fig 3C). GO analysis of the upregulated genes by LPS showed that the biological functions (GO terms) were related to lipid, protein, and redox metabolism (Fig 3D). GO analysis showed that biological pathways suppressed by MIC-1 were associated with immune system and inflammatory responses in the skeletal muscle tissue (Fig 3D; S2 File). These results support the hypothesis that MIC-1 attenuates the LPS-induced inflammation in the skeletal muscle tissue.

### MIC-1 inhibits LPS-induced acute inflammation in mice

**Fig 4:**
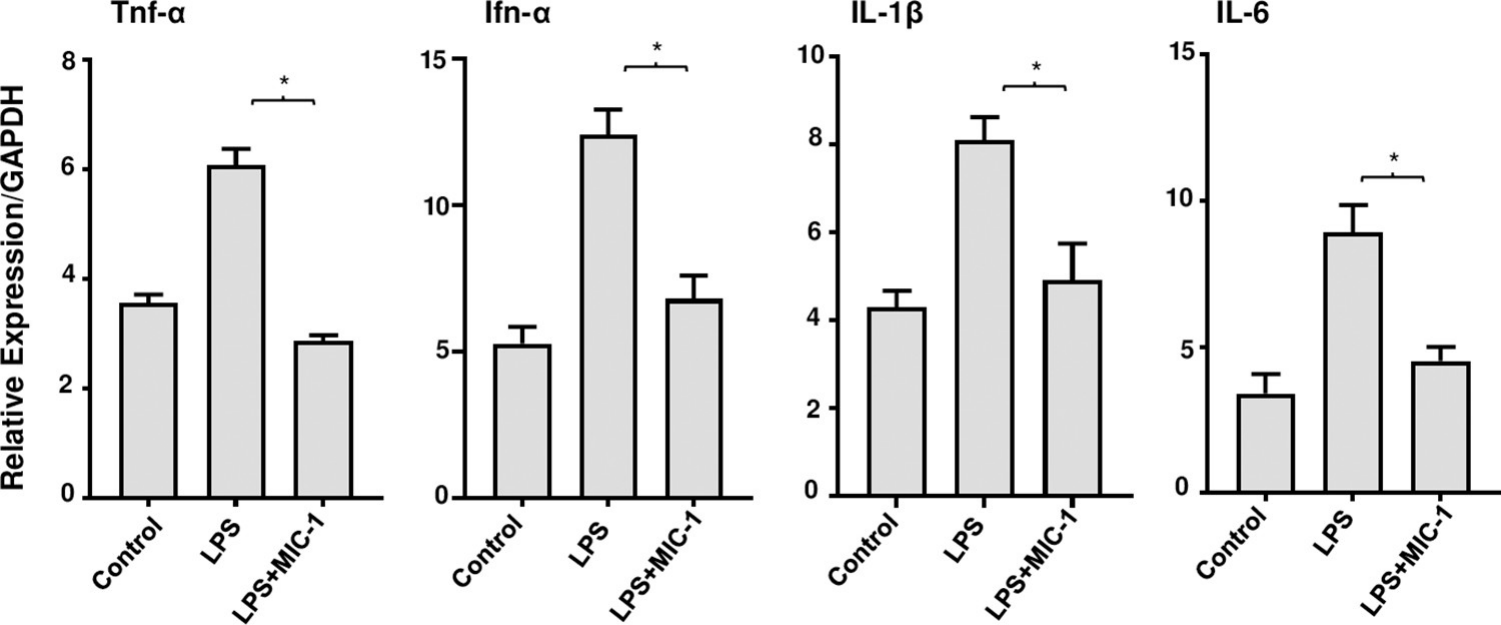
Moringa isothiocyanate-1 (MIC-1) reduced lipopolysaccharide (LPS)-induced pro-inflammatory markers in muscle tissue. TNF-α, Ifn-α, IL-1β, and IL-6 mRNA levels in the gastrocnemius muscle tissues treated with LPS and/or MIC-1 were measured by RT-qPCR. Data are represented as mean ± S.D. (n = 3). * *P* < 0.05, obtained by t-test when compared to LPS-treated group.

To validate the transcriptomic analyses, we evaluated the effects of MIC-1 treatment on the pro-inflammatory cytokines in murine muscle tissues by RT-qPCR. As expected, our results showed that MIC-1 decreased LPS-induced gene expressions of TNF-α, Ifn-α, IL-1β, and IL-6 in muscle tissue (Fig 4). These results corroborated with our transcriptomic analysis by RNA-Seq data showing the suppression of inflammatory cascades by MIC-1.

### MIC-1 inhibited the NF-κB nuclear translocation in skeletal muscle cells

**Fig 5:**
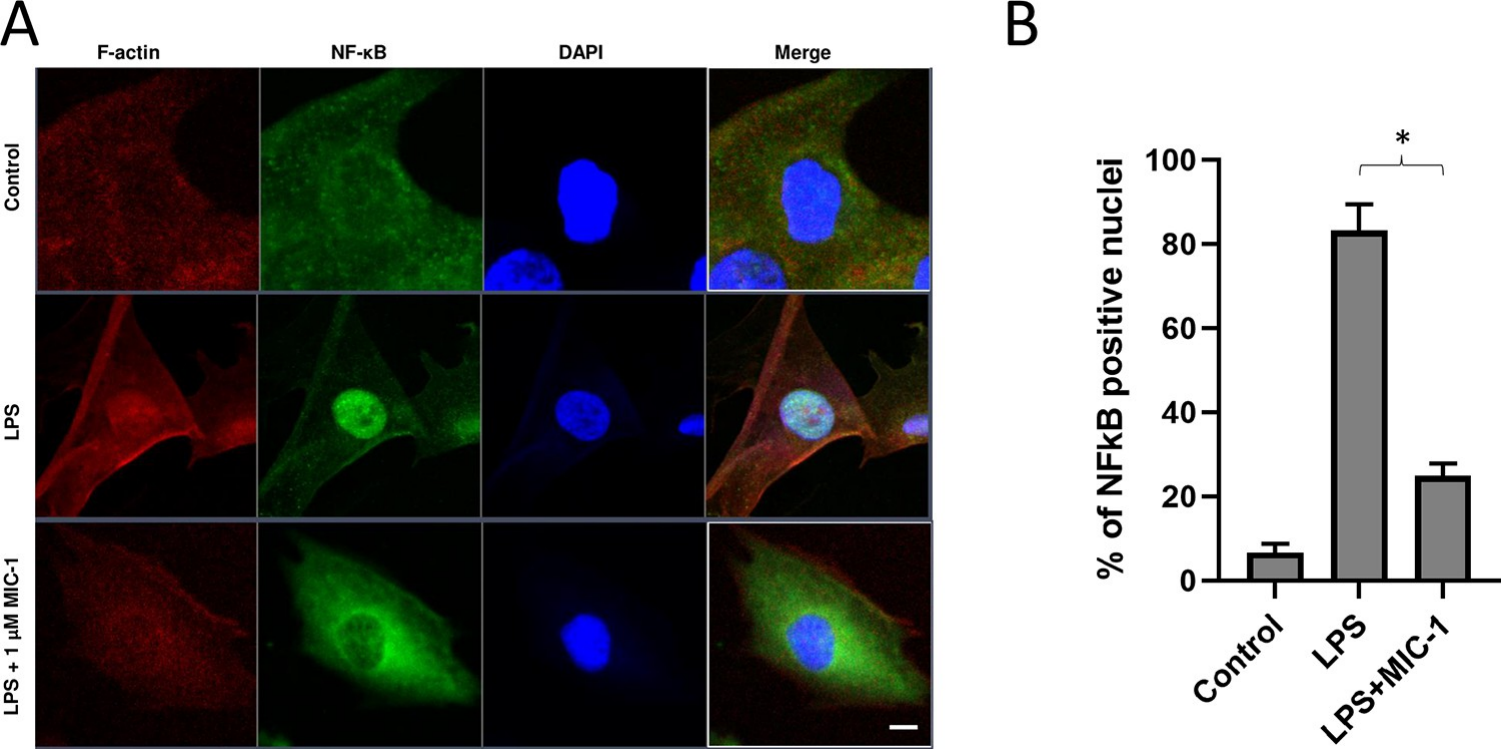
Moringa isothiocyanate-1 (MIC-1) inhibited the NF-κB nuclear translocation in skeletal muscle cells. The effects of MIC-1 on lipopolysaccharide (LPS)-induced nuclear translocation of NF-κB in C2C12 muscle cells. Treatments: untreated control, LPS (10 μg/mL), and LPS + 1 μM MIC-1 for 1 h. The NF-κB (p65) location was observed using FITC-anti-NF-κB p65 antibody, phalloidin-actin (F-actin; cytoskeleton), and DAPI (nuclei). (A) Representative pictures taken by using confocal microscopy. Scale bar: 20 μm. (B) The percentage of nucleic positive cells to NF-κB (p65). Data are represented as mean ± SD (n = 6, from three independent experiments). * *P* < 0.05, obtained by t-test when compared to LPS-treated group.

NF-κB is transcription factor that regulates pro-inflammatory cytokines, and our previous study showed that MIC-1 inhibited the nuclear translocation of NF-κB in macrophages [8]. To test whether MIC-1 inhibits nuclear translocation of NF-κB, we performed immunofluorescence staining of NF-κB in C2C12 myoblasts. Confocal microscope imaging showed that LPS increased the nuclear translocation of NF-κB, which was inhibited by 1 μM MIC-1 in an hour (Fig 5). These results further confirm that the inhibition of NF-κB pathway is part of the mechanisms of action of MIC-1 on inflammation in skeletal muscle.

## Discussion

MIC-1 is a chemically stable anti-inflammatory isothiocyanate from *M*. *oleifera* seeds. Our previous studies documented that MIC-1 activates anti-inflammatory processes in macrophages by inhibiting the NF-κB pathway [2, 7, 8]. However, the mechanisms of the anti-inflammatory action of MIC-1 in skeletal muscles have not been investigated. Since inflammation is an underlying cause of multiple muscle disorders, this study uses global transcriptomic analyses, selective gene expression analyses, and immunofluorescence staining to elucidate MIC-1’s anti-inflammatory mechanisms using the LPS-induced inflammation models in murine C2C12 myoblasts and skeletal muscle tissue. Our results showed that MIC-1 suppressed the LPS-induced inflammation by decreasing the nuclear translocation of NF-κB in the skeletal muscle cells. MIC-1’s anti-inflammatory properties were confirmed in skeletal muscle of mice, suggesting that the oral administration of MIC-1 prevents systemic inflammation.

LPS, a membrane component of Gram-negative bacteria, is used to induce inflammation and sepsis in pre-clinical studies [1, 2, 7, 8, 10–12, 25]. The induced innate immune response is initiated by recognizing LPS via Toll-like receptors (TLR), which activate NF-κB pathway inducing the expression of cytokines (e.g., TNF-α, IL-6 and IL-1β) to protect against infections [26, 32, 33]. Our data confirmed that LPS increased pro-inflammatory markers, such as cytokines and NO production in murine muscle. Our data also confirmed that the proliferating myoblasts are responsive to LPS-induced inflammation in a manner similar to differentiated myotube cells as previously reported by others [27]. It is also known that LPS can change protein and lipid metabolism and these metabolic changes occur before inducing an immune response in skeletal muscle [26, 32]. Others suggest that the LPS-induced metabolic changes, including immune response, are dependent on skeletal muscle type and exposure time [32–34]. Although in the present study we collected only one type of murine skeletal muscle (gastrocnemius, a fast-twitch glycolytic skeletal muscle) at one time point, we consistently showed that LPS treatment induces the upregulation of inflammatory markers in the skeletal muscle.

NF-κB is a transcription factor that regulates innate and adaptive immune responses mediated by inflammation. As a result, NF-κB activation contributes to the etiology of various inflammatory diseases. Our results showed that MIC-1 reduced the LPS-induced inflammation, at least partially, via NF-κB pathway in skeletal muscle. MIC-1 decreased the NF-κB’s nuclear translocation, as visualized by immunofluorescence staining. This observation may be validated further with cell fractionation experiments [35]. The observed MIC-1’s anti-inflammatory effects in skeletal muscle cells were similar to its effects in macrophages, which suggest that the oral administration of MIC-1 affects multiple inflammation-mediated processes [8]. Sulforaphane from Brassicaceae plants is the best studied natural isothiocyanate, however, in contrast to MIC-1 it is a chemically unstable liquid at room temperature (2). Similarly to MIC-1, sulforaphane also reduced inflammatory pathways in skeletal muscle [17–20]. Sulforaphane inhibited the NF-κB pathway followed by the downregulation of the inflammatory cytokines (TNF-α, IL-1β, IL-6) in skeletal muscle of mdx mice, a genetic model for muscle dystrophy [17]. Moreover, sulforaphane regulated the NF-κB’s upstream regulators by increasing the inhibitor of κB (IκB) and decreasing IκB kinase (IKK) at protein levels in skeletal muscle of mdx mice [17]. IKK is responsible for the phosphorylation of IκB releasing NF-κB into the nucleus, which suggests that sulforaphane inhibited NF-κB’s nuclear translocation by regulating its cytoplasmic content [17]. MIC-1 exerts its anti-inflammatory properties by inhibiting NF-κB pathway, however whether MIC-1 inhibits NF-κB pathway post-translationally via IKK/IκB is not yet known. Further studies may be needed to evaluate the effects of MIC-1 on specific NF-κB isoforms and related molecules, as well as to confirm NF-κB involvement using inhibitors or loss of function mutations.

MIC-1 may also regulate inflammation via other pathways, such as interferons (Ifn) signaling pathway. MIC-1 downregulated the expression of a type I interferon (Ifn-α), which attracts immune response cells to the infected areas. Interferons can induce immune response independently of NF-κB pathway, for example via activation of the Janus kinase (JAK)/signal transducer and activator of transcription (STAT) signaling pathway [36, 37]. Although sulforaphane reduced the phosphorylation of JAK2 and STAT3 in liver and adipose tissues of high fat diet-fed rats [36], sulforaphane and MIC-1 inhibited interferon-stimulated genes without regulating the phosphorylation of STATs in Ba/F3 lymphocytes and HeLa epithelial cells [37]. MIC-1 and sulforaphane may inhibit JAK/STAT pathway by downstream inhibitory events or other posttranslational modifications [37]. In addition, previous reports suggest that sulforaphane reduces interferon response by downregulating the stimulator of interferon genes (STING) via the nuclear factor erythroid 2–related factor 2 (Nrf2) pathway [38, 39]. We previously reported that MIC-1 increases the nuclear translocation of Nrf2, followed by a reduction of oxidative stress and inflammation markers induced by LPS in macrophages [8]. Consistently, moringa leaf extract increased the activities of Nrf2 target enzymes (i.e., superoxide dismutase and catalase) in C2C12 myotubes [22, 24]. Therefore, MIC-1’s anti-inflammatory properties may also include JAK/STAT, and STING/Nrf2 pathways. Other pathways identified by our GO analyses (S1 and S2 Files) may also be involved in the MIC-1’s anti-inflammatory effects in skeletal muscle, however how these pathways interact due to MIC-1 needs further investigation. We did not evaluate the effects of MIC-1 alone (without LPS induction) in muscle cells. However, we previously reported that MIC-1 showed similar anti-inflammatory effects in murine and human macrophages with and without LPS induction [1, 8].

The effective and safe dosages of MIC-1, given orally, used in rodent models can be translated to 6.5–16 mg/kg bw (390–960 mg/60 kg bw) in human equivalent doses [4, 8, 40]. However, clinical trials are yet to show MIC-1’s effectiveness and safety in humans. The clinical trials that investigated the effects of sulforaphane on inflammatory markers are still not definitive [18, 41]. While sulforaphane (30 mg/d for 4 weeks) reduced exercise-induced IL-6 levels in young healthy men [18], sulforaphane (up to 26.6 mg/d for 4 weeks) did not change inflammatory markers, including IL-6 levels, in patients with chronic obstructive pulmonary disease [41]. Pre-clinical studies showed that MIC-1 and sulforaphane have similar biological properties, but the anti-inflammatory effects of isothiocyanates in humans still need to be elucidated. In summary, MIC-1 suppresses the inflammatory pathways and pro-inflammatory cytokines induced by LPS in skeletal muscle. Overall, our study suggests that MIC-1 is a potential intervention to reduce systemic inflammation, inflammation-associated metabolic, and muscular dysfunctions.

## Supporting information

S1 Fig Principal component analysis (PCA) plots of RNA-seq data. DESeq2 analysis of the 3 replicates for C2C12 samples (A) and muscle tissue samples (B) was conducted to create PCA plots. The data were first turned into a DESeq2 object, then ’regularized log’ transformed and plotted in a PCA plot.
(TIF)

S1 File Gene Ontology (GO) analysis of C2C12 myoblasts. Gene counts, gene list, and GO terms of comparisons between control (Ctrl) vs lipopolysaccharide (LPS)-treated samples, and LPS-treated samples vs LPS-moringa isothiocyanate 1 (MIC-1)-treated samples.
(XLSX)

S2 File Gene Ontology (GO) analysis of gastrocnemius muscle of C57BL/6 male mice. Gene counts, gene list, and GO terms of comparisons between control (Ctrl) vs lipopolysaccharide (LPS)-treated samples, and LPS-treated samples vs LPS-moringa isothiocyanate 1 (MIC-1)-treated samples.
(XLSX)
