# Supplementary figures and images for: Moringa isothiocyanate-1 inhibits LPS-induced inflammation in mouse myoblasts and skeletal muscle
Source: PLoS One. 2022 Dec 16;17(12):e0279370. doi: 10.1371/journal.pone.0279370 (PMC9757596; doi:10.1371/journal.pone.0279370)

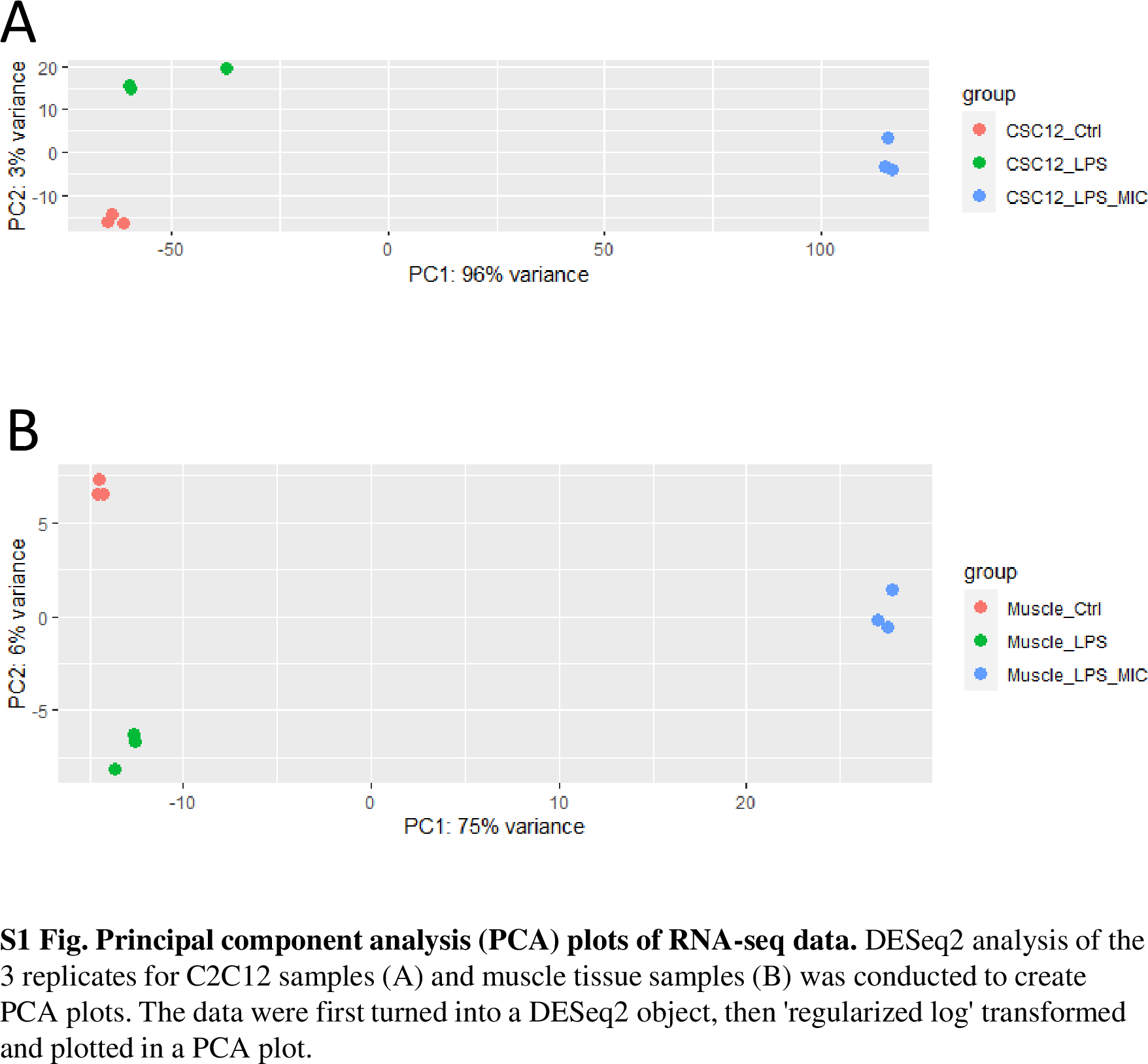

Supplement: S1 Fig — DESeq2 analysis of the 3 replicates for C2C12 samples (A) and muscle tissue samples (B) was conducted to create PCA plots. The data were first turned into a DESeq2 object, then ’regularized log’ transformed and plotted in a PCA plot. (TIF) [file pone.0279370.s001.tif]
